# Dynamics of changes in apical cell area during sex comb rotation in *Drosophila melanogaster.*

**DOI:** 10.17912/micropub.biology.000668

**Published:** 2022-12-13

**Authors:** Aaron Doerksen, Megan Mulder, Stephen Ingram, David Nelson, Jasmine Defehr, Eleanor Reimer, Joel Atallah, Juan Nicolas Malagon

**Affiliations:** 1 Canadian Mennonite University, Winnipeg, MB, Canada; 2 University of Waterloo, Waterloo, ON, Canada; 3 University of New Orleans, New Orleans, LA, USA

## Abstract

Epithelia are highly dynamic tissues displaying various types of tissue rearrangements (Weliky and Oster, 1990; Taylor and Adler, 2008; Harris and Tepass, 2010; Lee *et al.* , 2013; Firmino *et al.* , 2016; Rupprecht *et al.* , 2017). Here, we describe the dynamics of changes in apical cell area (ACA) in an epithelial system displaying tissue rearrangement resulting in sex comb rotation on the forelegs of male *Drosophila melanogaster* .

The sex comb is a row of leg bristles which rotates during morphogenesis (Atallah, 2008; Atallah *et al.* , 2009; Malagon, 2013). We quantified the ACA in the region proximal to the developing sex comb by tracing apical cell boundaries using *ImageJ* in pupal first leg imaginal discs. We found that cells display intricate irregular oscillations in size as the comb rotates. However, the net changes in ACA within most of the cells studied are subtle, only 0 to +/-15%. Our current working hypothesis suggests these irregular oscillations confer flexibility during tissue rearrangement and can be an important mechanism for tissue homeostasis.

**Figure 1. Changes in apical cell area (ACA) during sex comb rotation in Drosophila melanogaster:**
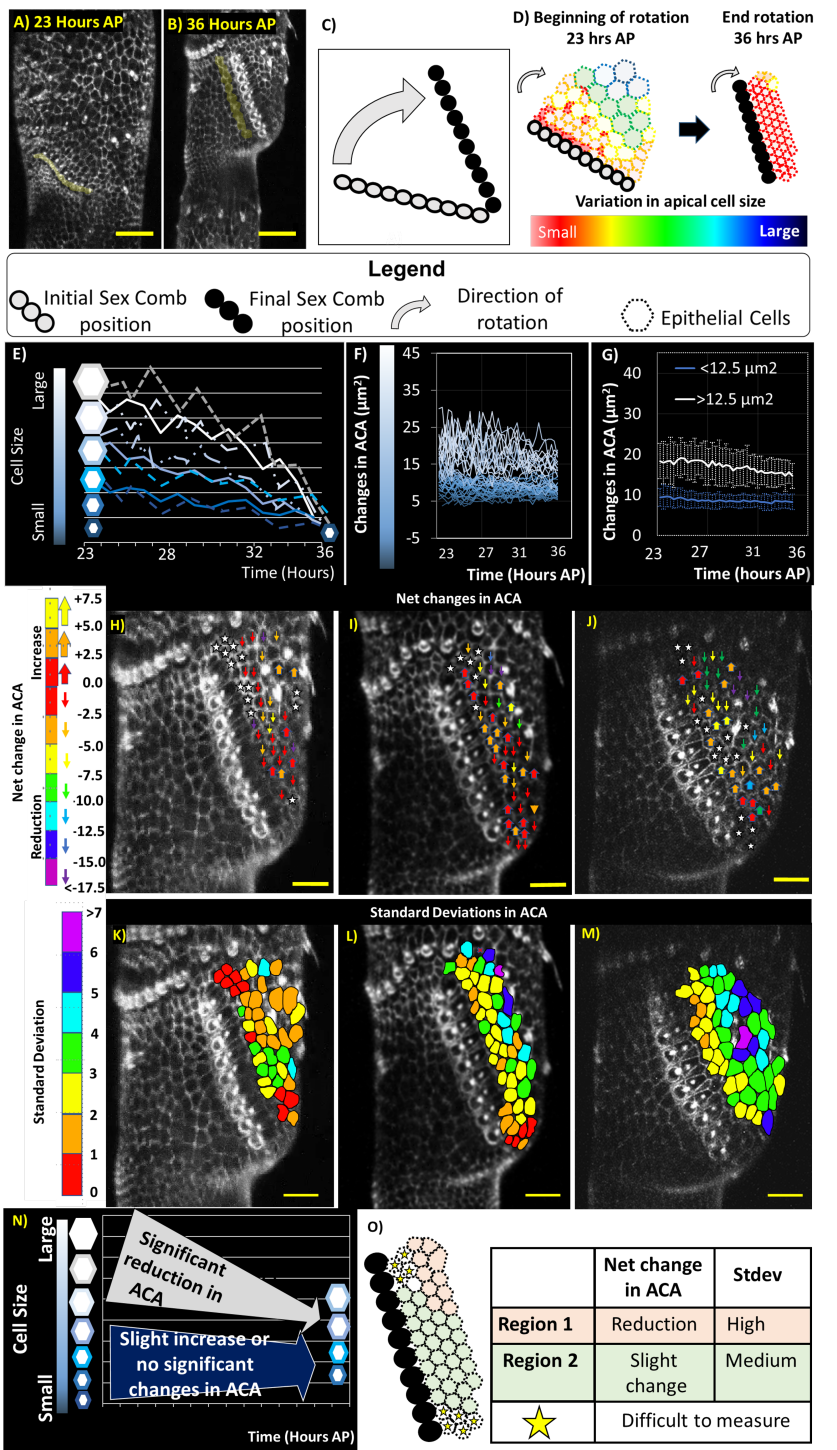
A-B) The 2D projections of different stages of the sex comb rotation process in *D. melanogaster* (developing sex comb cells shaded in yellow. C). Schematic diagram showing the rotation of sex comb from the initial diagonal position (grey circles) to its final vertical position (black circles). D) Schematic displaying the reduction in variation cell size that occurs during rotation. Above the sex comb, the epithelial cells become more homogeneous in cell size during the rotation. E) Schematic displaying the convergence in apical cell size during comb rotation. Our working hypothesis is that epithelial cells display relatively similar cell dynamics to reduce in ACA. F) Temporal patterns in changes in ACA of movie 1. G) Average of temporal changes in ACA of movie 1. H-J) Fate maps of net changes in ACA during comb rotation. K-L) Fate map of standard variations in ACA during comb rotation. Schematic summarizing temporal patterns (N) and spatial patterns (O) in ACA. Scale bar = 20 μm. After pupation = AP.

## Description

Epithelia are sheets of interconnected cells, which are fundamental tissue structures in animal morphogenesis (Harris and Tepass, 2010). In recent years, the study of epithelial morphogenesis has incorporated the use of live imaging (Weliky and Oster, 1990; Taylor and Adler, 2008; Lee *et al.* , 2013; Firmino *et al.* , 2016; Rupprecht *et al.* , 2017), showing that epithelia are highly dynamic tissues, requiring the interaction of multiple cellular processes including exchanging of cell neighbors (Taylor and Adler, 2008), changing in cell number, size and shape (Marinari *et al.* , 2012; Moreno *et al.* , 2019). However, despite the rapid progress in this field, it is still unknown how epithelia coordinate and buffer tissue rearrangement, while maintaining its functions as a physical barrier.

An excellent model system for studying the dynamics of tissue rearrangement is sex comb rotation in *Drosophila melanogaster* (Malagon, 2013). The sex comb is a linear row of modified bristles located on the forelegs of *D. melanogaster* males (Atallah *et al.* , 2009; Kopp, 2011). During pupation, the sex comb rotates from a perpendicular to a parallel position relative to the leg’s long axis (Atallah *et al.* , 2009; Kopp, 2011) (Fig. 1A-B). Previous work has shown that as the comb rotates, epithelial cells proximal to the sex comb rearrange displaying cell extrusion, exchange of cell neighbors, and changes in apical cell area. Here, we report that intricate irregular oscillations in cell size take place during tissue rearrangement. This variation in cell size during development can confer flexibility for tissue rearrangement without affecting epithelial integrity as a continuous barrier.

To investigate the dynamics of changes in apical cell area *,* we imaged wild type *D. melanogaster* legs using the line Ubi *D* Ecadherin::GFP which outlines cell boundaries (Oda and Tsukita, 2000). Then we manually outlined each cell through time using the imaging software, *ImageJ.* Later, we measured the changes in apical cell area (ACA) in the cells proximal to the sex comb (Malagon, 2013). Previous work showed that from a tissue perspective, the proximal region reduces to 1/2 of its original apical from cell rearrangement and elimination which results in comb rotation (Malagon, 2013) (Fig.1D). In addition, similar to other epithelial systems displaying tissue rearrangement via cell elimination, the epithelium proximal to the sex comb becomes more homogeneous in cell size ^15^ (Fig. 1D).

We found that although convergence in cell size occurs as previously reported (Malagon, 2013) (Fig. 1D-E), the cell dynamics to achieve tissue homogeneity display more complex temporal patterns than expected (Fig. 1F). In the three movies analyzed, cells display intricate irregular oscillations in ACA (Fig. 1F and Mov. 2 and 3 are shown in Fig. S1). However, we found a different behavior between large and small cells. Large cells (>12.5 μm ^2^ ) are clearly reducing in size (Average change in ACA Mov. 1=-3.69 μm ^2^ , Mov. 2=-4.08 μm ^2^ , Mov. 3=-3.4 μm ^2^ ). In contrast, small cells (<12.5 μm ^2^ ) tend to remain a similar size or display a slight increase in ACA (Average change Mov. 1= -1.14 μm ^2^ , Mov. 2= 1.35 μm ^2^ , Mov. 3=2.55 μm ^2^ ). In addition, large cells also display a higher variation in cell size (Standard deviation in ACA = Mov. 1= 4.38 μm ^2^ . Mov. 2 = 5.05 μm ^2^ , Mov. 3 = 5.60) than small cells (Standard deviation in ACA = Mov. 1= 2.00, Mov. 2 = 3.47, Mov. 3 = 3.51).

To dissect this intricate pattern in more detail, we constructed the following two types of fate maps: 1) net changes in ACA (Fig. 1H-J) and 2) variation in ACA of each cell during rotation (Fig. 1K-L). In both types of fate maps, there are some commonalities as well as differences among movies. In particular, most of the differences are found in cells close to cells of individual bristles. The highest values of net reduction in ACA and standard variation are found in the cells located farthest from the sex comb (Fig. 1H-J). In addition, close to the sex comb, changes in ACA are generally very subtle (Fig. 1H-J). However, there is not a linear correlation between net changes in cell size and its variation during development (R ^2^ Mov. 1=0.37, Mov. 2 =0.30, Mov. 3=0.27). The smallest cells tend to have intermediate values of standard variation (Fig. S3).

Altogether, these findings in the dynamics ACA are consistent with previous studies in tissue rearrangement. For example, the complex patterns observed in the changes in ACA were also observed in other cellular processes such as exchange of cell neighbors (Levayer, Hauert and Moreno, 2015) and cell death (Marinari *et al.* , 2012; Malagon, 2013). Our current working hypothesis is that many cells display a small net change in ACA; however, a high variation in cell size can offer flexibility to the tissue to selectively eliminate cells without opening a gap in the epithelia. Future studies will need to test whether intricate patterns in apical size also take place in other systems, and its implications for tissue homeostasis and cancer formation.

## Methods

Three time-lapse videos of wild type male *D. melanogaster* were recorded using confocal microscopy and analyzed in this study following the protocol described by Atallah (2008) ^15^ . For live imaging, pupae were mounted in halocarbon oil (series 700; Halocarbon Products) on a coverslip (Sigma) and imaged with a laser 510 scanning confocal microscope (ZEISS model) at 25 degrees with a 40× objective, using LSM Browser software (ZEISS). Z-stacks had a 3 μm step size.

Analysis of these three videos was performed using the *ImageJ* software (NIH, http://rsb.info.nih.gov/ij). 2D projections were generated, then epithelial cells proximal to the sex comb were manually outlined and followed through the time of 23 to 36 hours after pupation (AP) (Fig. S3). We labelled more than 40 cells per movie (Mov. 1=44 cells, Mov. 2 = 51, Mov. 3 =55), depending on how easy it was to accurately label cells during morphogenesis. If cells were not easy to distinguish, we did not collect data for that specific time point. Cells that were eliminated during tissue rearrangement were not included in the analysis. The changes in the apical cell area were measured using the subroutine “Analyze Particles” function of the *ImageJ* software (NIH, http://rsb.info.nih.gov/ij ). Error bars represent +/- 1 standard deviation.

**Fate maps**

The net changes in ACA were calculated by subtracting the final time point (36 hours AP) from the initial time point (23 hours AP). The variations in ACA were calculated using the standard deviations.

## Reagents

**Fly strains**

The ubi- *D* Ecad::GFP lines were generated by Oda and Tsukita (2000), and I obtained copies from the Ulrich Tepass laboratory. Flies were reared on a yeast-cornmeal-molasses medium at 25°C.

## Extended Data

Description: Video. Time lapse movie of the sex comb rotation in Drosophila melanogaster. Scale bar = 20 μm.. Resource Type: Image. DOI: 10.22002/x2zq8-qpm13
